# Chytrid fungi shape bacterial communities on model particulate organic matter

**DOI:** 10.1098/rsbl.2020.0368

**Published:** 2020-09-23

**Authors:** Cordelia Roberts, Ro Allen, Kimberley E. Bird, Michael Cunliffe

**Affiliations:** 1 Marine Biological Association of the UK, The Laboratory, Citadel Hill, Plymouth, UK; 2 School of Biological and Marine Sciences, University of Plymouth, Plymouth, UK

**Keywords:** bacteria, fungi, particulate organic matter

## Abstract

Microbial colonization and degradation of particulate organic matter (POM) are important processes that influence the structure and function of aquatic ecosystems. Although POM is readily used by aquatic fungi and bacteria, there is a limited understanding of POM-associated interactions between these taxa, particularly for early-diverging fungal lineages. Using a model ecological system with the chitin-degrading freshwater chytrid fungus *Rhizoclosmatium globosum* and chitin microbeads, we assessed the impacts of chytrid fungi on POM-associated bacteria. We show that the presence of chytrids on POM alters concomitant bacterial community diversity and structure, including differing responses between chytrid life stages. We propose that chytrids can act as ecosystem facilitators through saprotrophic feeding by producing ‘public goods’ from POM degradation that modify bacterial POM communities. This study suggests that chytrid fungi have complex ecological roles in aquatic POM degradation not previously considered, including the regulation of bacterial colonization, community succession and subsequent biogeochemical potential.

## 1. Introduction

Particulate organic matter (POM) in aquatic ecosystems acts as ‘hotspots’ for bacteria [1–3] and fungi [4,5]. Microbial processing of POM has impacts on ecosystem functioning, including the biological carbon pump in the open ocean [6] and carbon transfer through freshwater food webs [7].

Bacteria–POM studies have characterized the microscale interactions between bacteria and particles, including the composition of colonizing communities [8–10], interactions between attached bacteria [11] and the dynamics of POM degradation [9–12]. Laboratory-based incubations with chitin microbeads as model POM have identified bacteria that colonize and degrade POM using extracellular enzymes, producing a pool of more freely available substrates, including dissolved organic matter (DOM), considered ‘public goods’ for other bacteria in the community to utilize [8].

Dikaryan fungi (Ascomycota and Basidiomycota) also attach to and degrade POM [13–18]. Given that POM-degrading fungi also use extracellular degradation mechanisms [19,20], it is likely that they produce ‘public goods’ for the wider community to exploit. Studies of freshwater leaf-degrading dikaryan fungi show that as bacteria lack key enzymes associated with plant polymer degradation [21], the production of low and intermediate weight DOM by fungi [22] may support enhanced bacterial growth on allochthonous leaf litter [21].

The roles of early-diverging saprotrophic fungal lineages, such as the Chytridiomycota (chytrids), in POM-associated processes are poorly understood. Chytrids are widespread fungi that produce motile zoospores to search for substrates to colonize, including allochthonous and autochthonous POM such as pollen [4], chitin-rich exuviae [23] and zooplankton carcasses [24], as well as living substrates, such as amphibian epidermises [25] and phytoplankton [26]. Once attached, a zoospore loses its flagellum before developing a walled sporangium with a rhizoid network, which attaches to and penetrates the substrate [27]. Chytrids subsequently feed saprotrophically via the rhizoids, which secrete extracellular enzymes to degrade POM to low molecular weight substrates for uptake and assimilation [28].

Even though chytrids and bacteria coexist in aquatic ecosystems, knowledge of chytrid–bacteria POM interactions is limited to niche overlap [4] and infection-associated dynamics on amphibian epidermises [29,30]. To our understanding, there is no current research on the direct influence of chytrids on POM-attached bacterial diversity and community structure. To address these knowledge gaps, we used the chitinophilic *Rhizoclosmatium globosum* in an experimental study with chitin microbeads to assess the interactions between chytrids, bacteria and POM. Using chitin microbeads as a POM experimental system removes the complex heterogeneity of natural particles (e.g. age and composition) while retaining ecological relevance since chitin is an important POM component in aquatic ecosystems [8]. We aimed to explore how the different chytrid life history stages (i.e. attaching zoospores versus established sporangia with rhizoid networks) impact concomitant attaching bacterial diversity and community structure.

## 2. Material and methods

### (a) Experimental set-up

*R. globosum* JEL800 was maintained on PmTG agar [31] as described previously [27]. To harvest zoospores, established plates were flooded with 4 ml distilled H_2_O and incubated at room temperature under laminar flow for 90 min. The zoospore suspension was passed through a 10 µm cell strainer and the concentration determined using a coulter Counter (Beckman Coulter, US).

**Figure 1.**
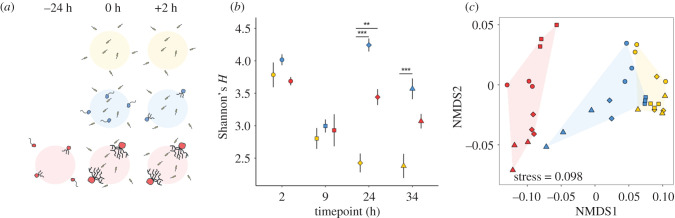
(*a*) Schematic summary of experimental design. Experimental treatments: Control = yellow, Zoospores = blue, Established = red. (*b*) Bacterial diversity measured as the Shannon's *H* index of treatments over time. Bars represent standard error and asterisks denote level of significance as follows: **p < 0.01, ***p < 0.001. (*c*) NMDS plot of bacterial community structure based on weighted UniFrac dissimilarity between bacterial communities found with treatment over time.

Magnetic chitin microbeads (New England Bio) were used for the experiments using protocols adapted from [8]. Pond water containing a natural bacterial assemblage was collected from Efford Marsh pond (Plymouth, UK) and passed through a 40 µm mesh to remove detritus and large eukaryotes. Three experimental treatments were set up as follows: ‘Control’, 40 µm filtered pond water and chitin microbeads; ‘Zoospores’, *R. globosum* zoospores, 40 µm filtered pond water and chitin microbeads; and ‘Established’, *R. globosum* grown initially on chitin microbeads for 24 h in 0.2 µm filtered pond water before addition of experimental 40 µm filtered pond water (figure 1*a*).

For each treatment, 23.5 ml pond water was added to a 25 ml vented culture flask. Each treatment was conducted with three replicate flasks per timepoint that were sampled destructively. Chitin microbeads were added to the flask and inverted several times to ensure even distribution. For chytrid treatments, zoospores were added to a concentration of approximately 3 × 10^4^ cells ml^−1^. Flasks were incubated in the dark at 22°C with mixing at 50 rpm. After 2 h, a washing step took place in the chytrid treatments, with the water replaced to ensure that the experiments proceeded with microbead-attached chytrids only (‘Established’ 40 ± 14.9 chytrids microbead^−1^ and ‘Zoospores’ 37 ± 15.8 chytrids microbead^−1^). After 2 (i.e. the wash step), 9, 24 and 34 h, all chitin microbeads from each flask were harvested using a magnet. The residual water was discarded, retaining the chitin microbeads, which were frozen in liquid nitrogen and stored at −80°C.

### (b) DNA extraction, 16S rRNA gene sequencing and bioinformatics

DNA was extracted from the chitin microbeads using the Zymo Research *Quick-*DNA™ Fecal/Soil Microbe Microprep Kit (Zymo Research, USA) following the manufacturer's instructions. The V4–V5 region of the 16S rRNA gene was amplified using primers 515FB and 926R [32], and sequenced using the Illumina MiSeq platform. Sequences were processed in R [33] using the DADA2 pipeline [34]. Demultiplexed reads were filtered and trimmed to remove primers and low-quality sequences. The DADA2 algorithm was used to infer amplicon sequence variants (ASVs) [34]. Paired-end reads were merged to obtain full denoised sequences. Chimeric sequences were removed, before taxonomy was assigned using the SILVA database (release 128) [35]. ASVs assigned as chloroplasts and mitochondria were removed. A maximum likelihood phylogenetic tree was estimated using the *phangorn* package (v.2.5.5) [36] and combined with the ASV table, taxonomic assignment and experimental metadata into a phyloseq object using the *phyloseq* package [37]. Sequences were rarefied to 4955 reads before further analysis.

### (c) Data processing and statistical analyses

Shannon's index (*H*) was used to calculate diversity, and the effect of treatment and time on diversity was assessed using a two-way ANOVA with Tukey's HSD. Differences in community structure between samples (beta diversity) were calculated using a weighted UniFrac [38] distance matrix and visualized through non-metric multidimensional scaling (NMDS) ordination. Permutational multivariate analysis of variance (PERMANOVA) [39] was used to test the effect of treatment and time on community structure using the ‘adonis’ function in the R package *vegan* [40].

## 3. Results

The diversity of the bacterial communities attached to chitin microbeads in all treatments followed the same pattern of decline in the first 9 h of the experiment (figure 1*b*). After 9 h, bacterial diversity in the control treatment continued to decrease for the remainder of the study. Conversely, bacterial diversity in the presence of chytrids increased after 9 h compared with the control treatment (Tukey's HSD *p* < 0.05). Attached bacterial diversity was greater in the zoospore treatment compared with the established chytrid treatment, although this trend was not statistically significant (Tukey's HSD *p* = 0.08).

**Figure 2.**
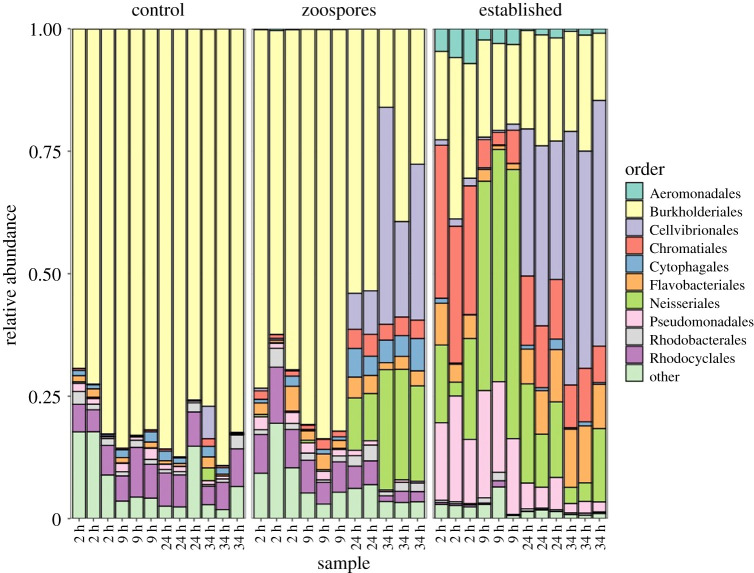
Bacterial community composition. Bacteria are grouped by the top 10 most abundant orders; orders outside this are grouped as ‘other’.

Bacterial community composition varied significantly between treatments and timepoints, with a significant interaction between these variables (PERMANOVA, all *p* < 0.001). After 2 h, the bacterial communities attached to the chitin microbeads in the established treatment were distinct from the other treatments (figure 1*c*), dominated by Burkholderiales, Chromatiales and to a lesser extent Neisseriales and Pseudomonadales (figure 2). Conversely, bacterial communities in the zoospore and control treatments were similar (figure 1*c*) and dominated by Burkholderiales (figure 2).

As the experiment progressed, the structure of the bacterial communities in the zoospore and established chytrid treatments converged, while bacterial communities in the control treatment remained distinct and with relatively limited variation through time (figure 1*c*). At 24 h, Cellvibrionales were also dominant in both the zoospore and established treatments, while Burkholderiales remained dominant in the control treatment (figure 2). Together, these results suggest that the presence of chytrids on chitin particles impacts both initial bacterial colonization and community succession, with the effects specific to the chytrid life stage.

## 4. Discussion

Understanding microbial–POM interactions has been largely dominated by bacteria-focused studies. Limited work has been conducted on fungi–bacteria–POM interactions, and the roles of early-diverging fungal lineages in these interactions remains unresolved. Here we show that chytrid fungi impact POM colonizing bacteria and community succession, suggesting that chytrids play a role in shaping POM microbial communities, with implications for carbon cycling in aquatic ecosystems.

Community diversity across all treatments initially declined, following previously reported patterns of bacterial colonizing communities on chitin microbeads [8], suggesting that early colonization here was also governed by known mechanisms (e.g. attachment ability). However, as the presence of the established chytrids with developed rhizoids showed an initially distinct bacterial community structure from the zoospore treatment, this suggests that life-stage dependent ecological interactions occur between chytrids and bacteria that govern particle colonization.

Chitin degradation can result in the release of DOM in aquatic systems [41,42]. Prior to the addition of the bacteria, when the chytrids attach to and degrade the chitin microbeads, they likely produce extracellular *N*-acetylglucosamine (NAG) as a potential ‘public good’. Bacteria, including NAG-using bacteria that are unable to degrade chitin (i.e. ‘cheaters’), may be supported by this pool of ‘public goods’. Community-wide bacterial growth on chitin does not necessarily require chitin degradation by all community members and can be stimulated by the utilization of secondary degradation products including NAG [43,44]. Previous experimental work has suggested that POM-derived DOM utilization involves a diverse assemblage of bacteria, with no single group dominating the consumption of NAG [45]. The initial structure of the colonizing bacterial community in the established treatment suggests that the bacteria present may be using a source of DOM, such as the proposed NAG pool, and that chytrids may play a role in DOM production from POM degradation in aquatic systems.

The temporal change in bacterial community composition may indicate the decline of the potential NAG pool, and a switch towards a chitin-degrading community at 24 h. As there is a similar switch in community composition seen in the zoospore treatment, this could have been stimulated, in part, by chitin degradation products from the chytrids acting as chemotactic signals for degraders [46,47]. Furthermore, *R. globosum* JEL800 rhizoids form grooves on the outer surface and penetrate chitin microbeads [27], modifying the POM structure and providing increased surface area for bacterial attachment and promoting chitin degradation. It is also possible that, because the chytrid cell wall also contains chitin, the differences in diversity between chytrid and control treatments could be due to an increased relative abundance of chitin-degrading bacteria using chitin from living chytrids or necromass. The predicted function of bacterial communities in this study approximated using Piphillin [48] provides support to this suggestion, showing a convergence in the established and zoospore treatments and divergence from the control treatment, which were distinct over time (electronic supplementary material, figure S1). Piphillin analysis predicted an initially elevated abundance of the NAG transporter gene in the established treatment only that declined over time, presumably as the NAG pool was depleted (electronic supplementary material, figure S2E). Subsequently, there was a predicted increase in chitinase and chitin-oligosaccharide transport genes [49] at 24 h in the established and zoospore treatments (electronic supplementary material, figure S2). Future studies should attempt to unravel the exact nature of the chytrid–bacteria interactions reported here, including the impacts of bacteria on saprotrophic chytrids and the direct assessment of bacterial function, such as through enzyme assays or metatranscriptomics.

POM degradation by microbes may also result in the indirect generation of diverse carbon substrates such as cell debris and metabolic by-products (e.g. organic acids) [8]. Enhanced chemical heterogeneity of these alternative carbon sources produced by both chytrids and bacteria could support the colonization of bacteria that use these products and drive the community dynamics reported here. As the colonization of these bacteria on the particle is likely to invoke competitive interactions, such as for space, the collective community function may diverge from a directly chitin-degrading community towards one that relies on secondary production, as shown in bacteria-only studies [8].

Chytrids have established roles in aquatic ecosystems, including parasitizing hosts and transferring resources via lipid-rich zoospores to higher trophic levels through the mycoloop [50]. Overall, our data indicate that independent of life stage, chytrids also influence the diversity and community structure of POM-colonizing bacterial communities. Increased diversity of bacteria associated with chytrids suggests that chytrids may produce DOM as a pool of ‘public goods’ supporting the growth of ‘cheaters’ and/or encouraging the chemotaxis of chitinolytic bacteria. The potential stimulation of bacterial chitin degradation by the presence of established chytrids, coupled with their own inherent degrading capability, implies that saprotrophic chytrids may have complex roles in regulating POM and DOM processing in aquatic ecosystems that are not yet considered.

## Supplementary Material

Supplementary Information

## Supplementary Material

Supplementary Figure 1

## Supplementary Material

Supplementary Figure 2

## Supplementary Material

Supplementary Table 1

## Data accessibility

Sequence data have been deposited at the European Nucleotide Archive under project number PRJEB37940.
