# Supplementary material for: Chytrid fungi shape bacterial communities on model particulate organic matter
Source: Biol Lett. 2020 Sep 23;16(9):20200368. doi: 10.1098/rsbl.2020.0368 (PMC7532721; doi:10.1098/rsbl.2020.0368)
Supplement: Supplementary Information [file rsbl20200368supp1.docx]

**Roberts et al Chytrid fungi shape bacterial communities on model particulate organic matter**

**Supplementary Information**

Functional gene prediction based on ASVs was performed using Piphillin [48] with a 99% sequence identity cut-off. Putative functions were assigned to sequences by allocating KEGG orthologs (KO) from the KEGG reference database (release October 2018). Differences in putative community function between samples were calculated using a Bray-Curtis dissimilarity matrix and visualised using NMDS ordination. PERMANOVA was then used to compare functional profiles. To determine potential genes that contributed most to the observed dissimilarity between the functional profiles, the similarity percentage (‘simper’) function in the R package vegan was used.

As with bacterial community structure, the predicted function of the bacterial communities varied between treatment and time point, with a significant interaction between these variables (PERMANOVA, all p<0.001; Supplementary Figure 1). The functional potential of bacterial communities was similar between all treatments after 2 hours (Supplementary Figure 1). Throughout the experiment, the functional potential of bacterial communities with chytrids (zoospores and established treatments) progressed along a similar trajectory, which became divergent from communities in the control treatment. After 34 hours, the functional potential of bacterial communities in the zoospore and established chytrid treatments were different from the control treatment.

Initially, KOs encoding chemotaxis associated genes and ABC transporters were abundant across all treatments (Supplementary Table 1). As the experiment progressed, other KOs increased in the bacterial communities with chytrids, including genes encoding the TonB-ExbBD complex (KO3832, KO3561, KO3559) (Tukey’s HSD p<0.05), which is involved in the trans-membrane transport of chitin-oligosaccharides (Supplementary Figure S2A and B-D). KOs for N-acetylglucosamine (NAG) transport (KO2804) and chitinase (KO11883) were also identified. The relative abundance of the NAG transporter gene was higher in the bacterial communities in the presence of the established chytrid initially (Tukey’s HSD p<0.001) and then declined at 24 hours to a similar level as the other treatments (Supplementary Figure 2E). Conversely, the relative abundance of the chitinase KO was initially low across all treatments, and then increased in the established chytrid treatment and subsequently in the zoospore treatment to similar levels (Tukey’s HSD p<0.01) (Supplementary Figure 2F).

**Supplementary Figure 1** NMDS plot of potential bacterial community function based on Bray-Curtis dissimilarity of KO abundances with treatment over time.

**Supplementary Figure 2** (A) Bacterial chitin degradation pathway adapted from [50]. Chitin is degraded by extracellular chitinases into chito-oligosaccharides that are transported across the outer membrane (O.M) using a specific chito-oligosaccharide channel, powered by the TonB-exbBD complex. In the periplasm, chito-oligosaccharides are degraded into N-acetylglucosamine and transported across the inner membrane (I.M), where they are further degraded within the cytoplasm. Predicted abundances for genes associated with the TonB-exbBD complex in control (yellow), zoospore (blue), and established (red) treatments. Predicted gene counts for genes (B) exbB, (C) exbD, (D) tonB, (E) NAG transporter and (F) chitinase normalized to the single copy housekeeping gene RNA polymerase (rpoB). Bars represent standard error and asterisks denote level of significance as follows; ‘*’ p <0.05, ‘**’ p <0.01, ‘***’ p <0.001.

**Supplementary Table 1** SIMPER analysis table of KEGG orthologs (KO) displaying the top 10 KOs contributing to differences between treatments over time.
