# Supplementary figures and images for: Chytrid fungi shape bacterial communities on model particulate organic matter
Source: Biol Lett. 2020 Sep 23;16(9):20200368. doi: 10.1098/rsbl.2020.0368 (PMC7532721; doi:10.1098/rsbl.2020.0368)

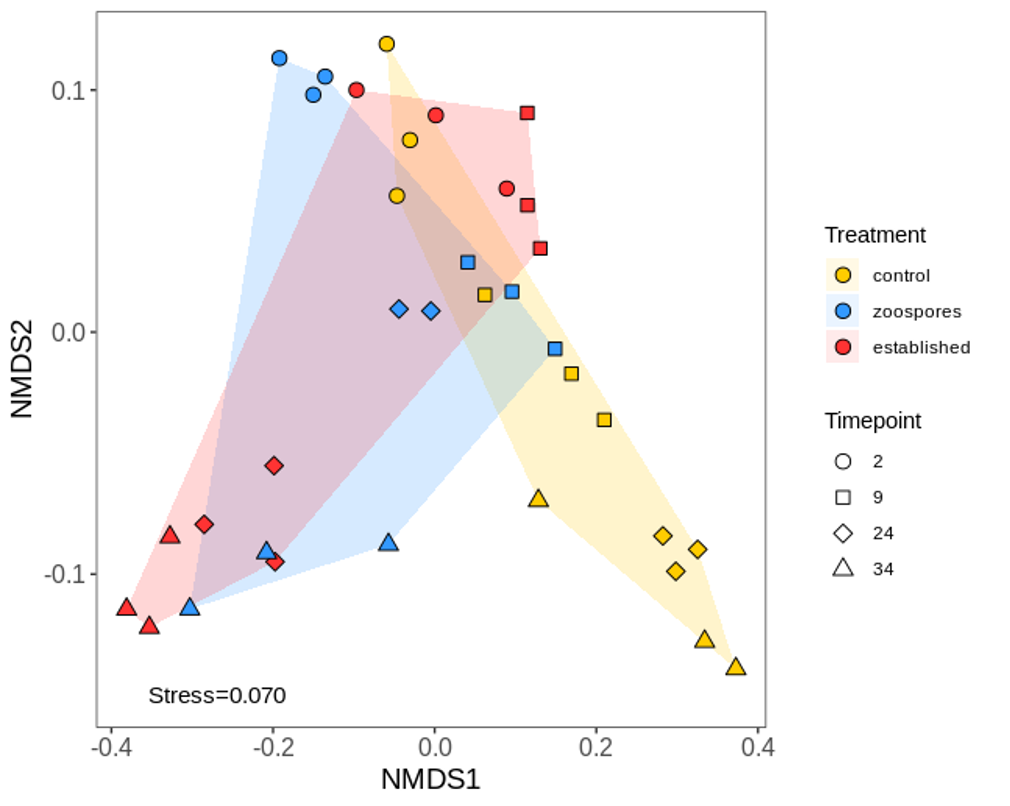

Supplement: Supplementary Figure 1 [file rsbl20200368supp2.tif]

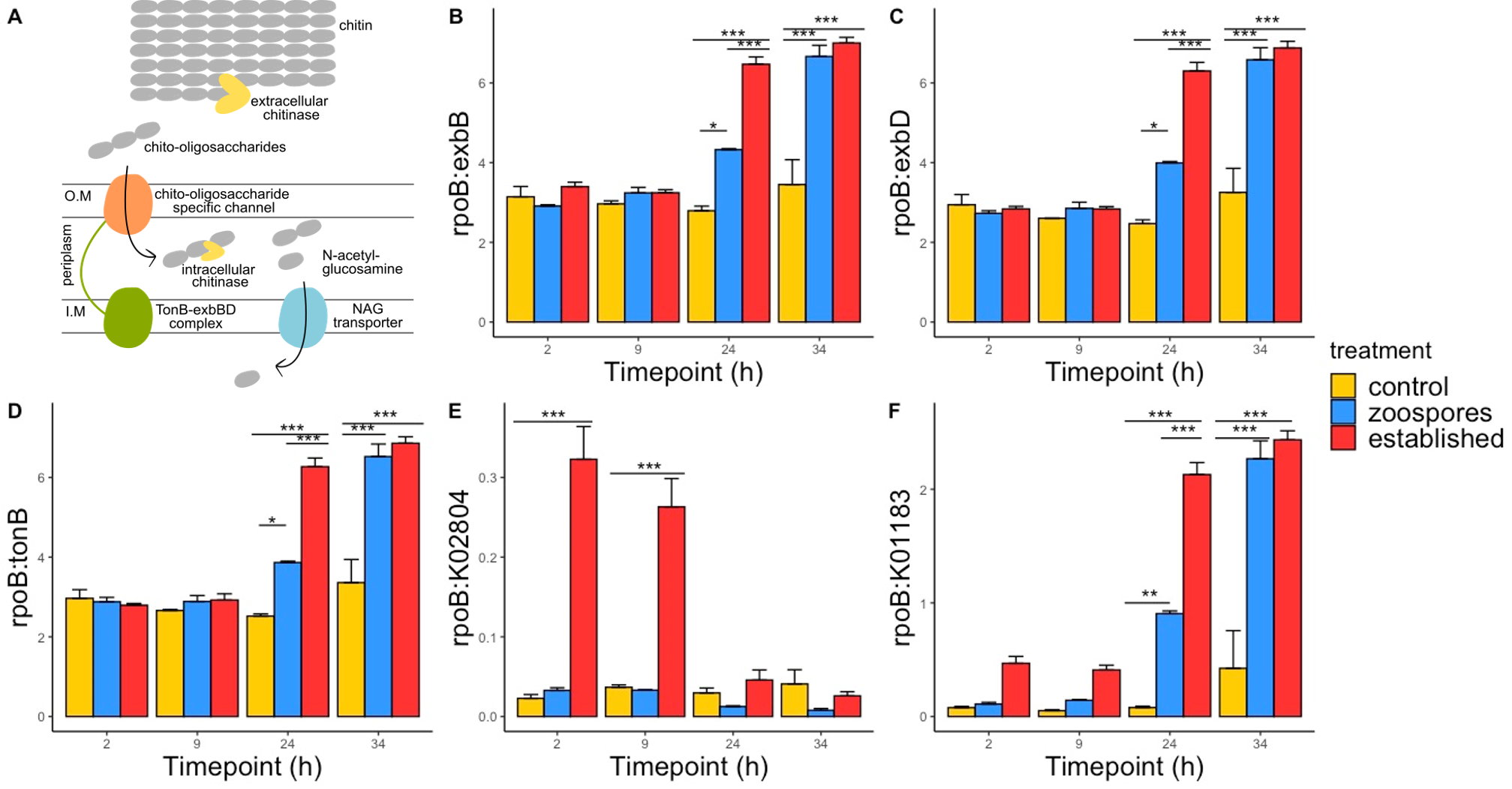

Supplement: Supplementary Figure 2 [file rsbl20200368supp3.tif]
