# Supplementary material for: Chytrid fungi shape bacterial communities on model particulate organic matter
Source: Biol Lett. 2020 Sep 23;16(9):20200368. doi: 10.1098/rsbl.2020.0368 (PMC7532721; doi:10.1098/rsbl.2020.0368)
Supplement: Supplementary Table 1 [file rsbl20200368supp4.pdf]

	Timepoint 2	SIMPER		
**KO**	Established vs just bacteria	contribution	mean abundance a	mean abundance b
**1999**	branched-chain amino acid transport system substrate-binding protein	0.0011286	4450.71	7626.02
**2030**	polar amino acid transport system substrate-binding protein	0.0010022	7040.35	4100.33
**3406**	methyl-accepting chemotaxis protein	0.0009277	10853.29	8752.6
**1997**	branched-chain amino acid transport system permease protein	0.0007867	3685.79	5887.96
**1995**	branched-chain amino acid transport system ATP-binding protein	0.0007786	3598.77	5776.45
**1998**	branched-chain amino acid transport system permease protein	0.000753	3827.53	5929.5
**1996**	branched-chain amino acid transport system ATP-binding protein	0.0007523	3721.87	5824.25
**2014**	iron complex outermembrane recepter protein	0.0007315	3946.99	5375.84
**3088**	rpoE; RNA polymerase sigma-70 factor,	0.0006238	3969.33	5454.65
**626**	atoB; acetyl-CoA C-acetyltransferase	0.000453	2901.17	4144.98
**KO**	Established vs zoospores	contribution	mean abundance a	mean abundance b
**1999**	branched-chain amino acid transport system substrate-binding protein	0.0011194	4450.71	8131.6
**7496**	K07496; putative transposase	0.0010893	426.26	4004.5
**3406**	methyl-accepting chemotaxis protein	0.0009877	10853.29	13567.5
**1998**	branched-chain amino acid transport system permease protein	0.0009078	3827.53	6815.2
**1995**	branched-chain amino acid transport system ATP-binding protein	0.0008975	3598.77	6550.5
**1997**	branched-chain amino acid transport system permease protein	0.00087	3685.79	6548.3
**1996**	branched-chain amino acid transport system ATP-binding protein	0.0008621	3721.87	6558.5
**626**	atoB; acetyl-CoA C-acetyltransferase	0.0007972	2901.17	5528.4
**2030**	polar amino acid transport system substrate-binding protein	0.0006489	7040.35	5080.3
**3496**	parA, soj; chromosome partitioning protein	0.0006148	1642.89	3673.2
**KO**	just bacteria vs zoospores v bac	contribution	mean abundance a	mean abundance b
**3406**	methyl-accepting chemotaxis protein	0.0014334	8752.6	13567.5
**7496**	K07496; putative transposase	0.0007758	1392	4004.5
**7481**	K07481; transposase, IS5 family	0.0005178	1604.4	3375.9
**2030**	polar amino acid transport system substrate-binding protein	0.0004852	4100.3	5080.3
**2014**	iron complex outermembrane recepter protein	0.0004671	5375.8	4994.1
**2004**	ABC.CD.P; putative ABC transport system permease protein	0.0004081	2694.9	4091.5
**626**	atoB; acetyl-CoA C-acetyltransferase	0.0004024	4145	5528.4
**3496**	parA, soj; chromosome partitioning protein	0.0003936	2328.9	3673.2
**1999**	branched-chain amino acid transport system substrate-binding protein	0.0003795	7626	8131.6
**2003**	ABC.CD.A; putative ABC transport system ATP-binding protein	0.0003746	2628.6	3909.8

Supplementary Table 1

	Timepoint 9	SIMPER		
**KO**	Established vs just bacteria	contribution	mean abundance a	mean abundance b
**3406**	methyl-accepting chemotaxis protein	0.0015076	7789.6	4983.909
**2014**	iron complex outermembrane recepter protein	0.0009686	3425.8	1608.445
**2030**	polar amino acid transport system substrate-binding protein	0.0007524	4223.9	2865.198
**3088**	rpoE; RNA polymerase sigma-70 factor,	0.0004983	3213.6	2410.049
**7496**	putative transposase	0.0004915	392	1330.444
**2029**	ABC.PA.P; polar amino acid transport system permease protein	0.0004428	2164.5	1348.946
**1999**	branched-chain amino acid transport system substrate-binding protein	0.0004077	3167.5	3892.704
**1998**	branched-chain amino acid transport system permease protein	0.0003645	2637.1	3349.012
**1996**	branched-chain amino acid transport system ATP-binding protein	0.0003547	2563.9	3250.502
**2073**	metQ; D-methionine transport system substrate-binding protein	0.0003517	889.1	230.534
**KO**	Established vs zoospores	contribution	mean abundance a	mean abundance b
**3406**	methyl-accepting chemotaxis protein	0.000846	7789.6	7028.32
**1999**	branched-chain amino acid transport system substrate-binding protein	0.0008206	3167.5	4874.41
**1998**	branched-chain amino acid transport system permease protein	0.0007202	2637.1	4132.75
**1996**	branched-chain amino acid transport system ATP-binding protein	0.0006924	2563.9	4001.95
**1997**	branched-chain amino acid transport system permease protein	0.0006916	2568.9	4005.63
**1995**	branched-chain amino acid transport system ATP-binding protein	0.000678	2524.5	3933.85
**7496**	K07496; putative transposase	0.0006399	392	1710.49
**2014**	iron complex outermembrane recepter protein	0.0005261	3425.8	2782.93
**626**	atoB; acetyl-CoA C-acetyltransferase	0.000344	2140.6	2861.15
**3088**	rpoE; RNA polymerase sigma-70 factor,	0.0003422	3213.6	3164.23
**KO**	just bacteria vs zoospores v bac	contribution	mean abundance a	mean abundance b
**3406**	methyl-accepting chemotaxis protein	0.001152	4983.9	7028.3
**2014**	iron complex outermembrane recepter protein	0.0006459	1608.4	2782.9
**1999**	branched-chain amino acid transport system substrate-binding protein	0.0006388	3892.7	4874.4
**2030**	polar amino acid transport system substrate-binding protein	0.0006191	2865.2	3913.7
**1998**	branched-chain amino acid transport system permease protein	0.000516	3349	4132.7
**1997**	branched-chain amino acid transport system permease protein	0.000503	3244.6	4005.6
**1996**	branched-chain amino acid transport system ATP-binding protein	0.0004982	3250.5	4001.9
**1995**	branched-chain amino acid transport system ATP-binding protein	0.0004946	3181.8	3933.9
**3088**	rpoE; RNA polymerase sigma-70 factor,	0.0004727	2410	3164.2
**799**	GST, gst; glutathione S-transferase [EC:2.5.1.18]	0.0003784	2413.8	3000.9

Supplementary Table 1

	Timepoint 24	SIMPER		
**KO**	Established vs just bacteria	contribution	mean abundance a	mean abundance b
**3406**	methyl-accepting chemotaxis protein	0.0064197	19641	2796.9728
**2014**	iron complex outermembrane recepter protein	0.0042629	12364	1177.9495
**3088**	rpoE; RNA polymerase sigma-70 factor,	0.0031072	10073	1909.3492
**14266**	prnA, rebH, ktzQ; tryptophan 7-halogenase	0.0030509	8048	52.4056
**1179**	Endoglucanase	0.0030354	8026	71.6389
**3561**	exbB; biopolymer transport protein ExbB	0.0021024	6307	791.6339
**3559**	exbD; biopolymer transport protein ExbD	0.0020747	6143	700.3865
**2004**	ABC.CD.P; putative ABC transport system permease protein	0.0020689	6624	1191.0452
**3832**	tonB; periplasmic protein TonB	0.0020575	6115	714.9624
**7483**	transposase	0.0016791	4986	577.7545
**KO**	Established vs zoospores	contribution	mean abundance a	mean abundance b
**3406**	methyl-accepting chemotaxis protein	0.0033183	19641	8224.6
**2014**	iron complex outermembrane recepter protein	0.0021164	12364	5080.1
**1179**	Endoglucanase	0.0017537	8026	2011.3
**14266**	prnA, rebH, ktzQ; tryptophan 7-halogenase	0.0017496	8048	2046.1
**3088**	rpoE; RNA polymerase sigma-70 factor,	0.0013891	10073	5275.9
**3832**	tonB; periplasmic protein TonB	0.0010638	6115	2454.9
**3559**	exbD; biopolymer transport protein ExbD	0.0010485	6143	2536.3
**3561**	exbB; biopolymer transport protein ExbB	0.0010345	6307	2746.8
**7483**	transposase	0.0008972	4986	1899.7
**2004**	ABC.CD.P; putative ABC transport system permease protein	0.0008771	6624	3594.5
**KO**	just bacteria vs zoospores v bac	contribution	mean abundance a	mean abundance b
**3406**	methyl-accepting chemotaxis protein	0.0028713	2796.973	8224.6
**2014**	iron complex outermembrane recepter protein	0.0020612	1177.949	5080.1
**2030**	polar amino acid transport system substrate-binding protein	0.0019734	1524.079	5252.5
**3088**	rpoE; RNA polymerase sigma-70 factor,	0.0017785	1909.349	5275.9
**1999**	branched-chain amino acid transport system substrate-binding protein	0.0014126	2274.556	4945.3
**1998**	branched-chain amino acid transport system permease protein	0.0012807	1941.526	4362.9
**2004**	ABC.CD.P; putative ABC transport system permease protein	0.0012696	1191.045	3594.5
**1996**	branched-chain amino acid transport system ATP-binding protein	0.0012607	1889	4273
**1997**	branched-chain amino acid transport system permease protein	0.0012457	1893.938	4249.4
**1995**	branched-chain amino acid transport system ATP-binding protein	0.0011839	1861.559	4100.4

Supplementary Table 1

	Timepoint 34	SIMPER		
**KO**	Established vs just bacteria	contribution	mean abundance a	mean abundance b
**3046**	methyl-accepting chemotaxis protein	0.0073426	28818	3809.6
**2014**	iron complex outermembrane recepter protein	0.0049204	18634	1864.1
**3088**	rpoE; RNA polymerase sigma-70 factor,	0.0037132	14709	2061.8
**1179**	Endoglucanase	0.0035699	12782	616.3
**14266**	prnA, rebH, ktzQ; tryptophan 7-halogenase	0.0035555	12794	676.3
**3561**	exbB; biopolymer transport protein ExbB	0.0024381	9395	1092.2
**2004**	ABC.CD.P; putative ABC transport system permease protein	0.0024269	9742	1476.7
**3559**	exbD; biopolymer transport protein ExbD	0.0024074	9231	1031.5
**3832**	tonB; periplasmic protein TonB	0.002391	9201	1057.4
**7483**	transposase	0.0020503	7736	747.1
**KO**	Established vs zoospores	contribution	mean abundance a	mean abundance b
**3046**	methyl-accepting chemotaxis protein	0.0024771	28818	17846
**2014**	iron complex outermembrane recepter protein	0.0015197	18634	11927
**3088**	rpoE; RNA polymerase sigma-70 factor,	0.0011946	14709	9419
**1179**	Endoglucanase	0.0011323	12782	7778
**14266**	prnA, rebH, ktzQ; tryptophan 7-halogenase	0.0011267	12794	7816
**3561**	exbB; biopolymer transport protein ExbB	0.0008014	9395	5845
**3832**	tonB; periplasmic protein TonB	0.0007838	9201	5733
**3559**	exbD; biopolymer transport protein ExbD	0.00078	9231	5780
**2004**	ABC.CD.P; putative ABC transport system permease protein	0.0007759	9742	6311
**7483**	transposase	0.0006349	7736	4931
**KO**	just bacteria vs zoospores v bac	contribution	mean abundance a	mean abundance b
**3406**	methyl-accepting chemotaxis protein	0.0056865	3809.6	17846
**2014**	iron complex outermembrane recepter protein	0.0040872	1864.1	11927
**3088**	rpoE; RNA polymerase sigma-70 factor,	0.0029944	2061.8	9419
**1179**	Endoglucanase	0.0029074	616.3	7778
**14266**	prnA, rebH, ktzQ; tryptophan 7-halogenase	0.0028968	676.3	7816
**2004**	ABC.CD.P; putative ABC transport system permease protein	0.0019655	1476.7	6311
**3561**	exbB; biopolymer transport protein ExbB	0.0019321	1092.2	5845
**3559**	exbD; biopolymer transport protein ExbD	0.0019289	1031.5	5780
**3832**	tonB; periplasmic protein TonB	0.001897	1057.4	5733
**7483**	transposase	0.0017007	747.1	4931

Supplementary Table 1
